# Preliminary exploration of the biomechanical properties of three novel cervical porous fusion cages using a finite element study

**DOI:** 10.1186/s12891-023-06999-2

**Published:** 2023-11-10

**Authors:** Zhi Xu, Yuwan Li, Weijun Huang, Ziru Wang, Xing Xu, Shoujin Tian

**Affiliations:** 1 Department of Orthopedic, Zhangjiagang Fifth People’s Hospital, Zhangjiagang, 215600 Jiangsu China; 2 Department of Orthopedic, Peking University Third Hospital, Beijing, 100191 China; 3 Department of Orthopedic, The First Affiliated Hospital, Zhejiang University School of Medicine, Hangzhou, 310000 China; 4 Department of Orthopedic, Shangyu Third Hospital, Shangyu, 312300 Zhejiang China; 5 Clinical Medical College, Wannan Medical College, Wuhu, 241000 Anhui China; 6 Department of Orthopedic, The First Affiliated Hospital of Wannan Medical College, Wuhu, 241000 Anhui China; 7 Department of Medicine, Zhijin People’s Hospital, Zhijin, 552100 Guizhou China; 8 Department of Orthopedic, Zhangjiagang First People’s Hospital, Zhangjiagang, 215600 Jiangsu China

**Keywords:** Anterior cervical discectomy and fusion, Topological optimization, Porous structure, Stress shielding, Additive manufacturing

## Abstract

**Background:**

Porous cages are considered a promising alternative to high-density cages because their interconnectivity favours bony ingrowth and appropriate stiffness tuning reduces stress shielding and the risk of cage subsidence.

**Methods:**

This study proposes three approaches that combine macroscopic topology optimization and micropore design to establish three new types of porous cages by integrating lattices (gyroid, Schwarz, body-centred cubic) with the optimized cage frame. Using these three porous cages along with traditional high-density cages, four ACDF surgical models were developed to compare the mechanical properties of facet articular cartilage, discs, cortical bone, and cages under specific loads.

**Results:**

The facet joints in the porous cage groups had lower contact forces than those in the high-density cage group. The intervertebral discs in all models experienced maximum stress at the C5/6 segment. The stress distribution on the cortical bone surface was more uniform in the porous cage groups, leading to increased average stress values. The gyroid, Schwarz, and BCC cage groups showed higher average stress on the C5 cortical bone. The average stress on the surface of porous cages was higher than that on the surface of high-density cages, with the greatest difference observed under the lateral bending condition. The BCC cage demonstrated favourable mechanical stability.

**Conclusion:**

The new porous cervical cages satifies requirements of low rigidity and serve as a favourable biological scaffold for bone ingrowth. This study provides valuable insights for the development of next-generation orthopaedic medical devices.

**Supplementary Information:**

The online version contains supplementary material available at 10.1186/s12891-023-06999-2.

## Introduction

The incidence of cervical spondylosis has been increasing annually. Although most patients demonstrate no clear symptoms, severe cases may cause symptoms of nerve roots or spinal cord compression, requiring surgical treatment. In 1955, Uschold et al. [1] created an anterior cervical discectomy and fixation operation (ACDF). At present, this operation has become the most commonly used surgical method for the treatment of various types of degenerative cervical diseases. In recent years, the use of intervertebral cages has become an indispensable part of ACDF surgery. It helps to achieve immediate postoperative stability, distract and maintain the height of the intervertebral space and has achieved satisfactory clinical results [2]. Nevertheless, with the increase in clinical surgical cases, complications directly related to cages often appear, and the most common complications are poor interface fusion and cage sinking [3]. Fusion dislocation caused by poor fusion of the surgical segment will cause pain, spinal cord and nerve root compression and other symptoms; fusion cage sinking will cause complications such as loss of intervertebral space height of the surgical segment and cervical spine instability, thus affecting the long-term curative efficacy and even leading to surgical failure, causing medical disputes. Therefore, how to reduce the incidence of such complications through the low stiffness design of cervical fusion cages has become an issue of concern in the field of spinal surgery.

Traditional titanium alloy cages have favourable mechanical strength, corrosion resistance and biocompatibility [4]. Clinical studies have shown that the high stiffness of the titanium cage leads to increased stress on the bone endplate and cage subsidence [5], and low elastic modulus cages have better fusion performance than high elastic modulus cages [6]. More recently, various strategies have been developed to reduce the stiffness of the cage, increasing the rate of intervertebral fusion while reducing the risk of cage subsidence. Topology optimization (TO) is a structural design technique that provides the optimal shape of a structure from a prescribed domain under specific design considerations such as loads and boundary conditions [7]. Guo et al. [8] redesigned an interspinous device using TO techniques and reported enhanced load transfer. Tamimi et al. designed a novel fracture fixation instrument that can reduce stress shielding through technology [9].

Porous titanium alloy can adjust its stiffness based on tuning the geometric parameters of the pore structure, so it is considered to be a promising candidate material for bone growth. The porous structure provides bone growth channels growth, and the integration between the porous structure and new bone increases local stability while reducing the risk of implant dislocation [10]. In addition, the porous scaffold is closer to the bone structure [11], and the porous structure can effectively reduce the elastic modulus, thus reducing the stress shielding effect [12]. The porous structure facilitates the transport of nutrients and promotes bone growth [13]. A three-periodic minimal surface (TPMS) is a type of minimal surface with periodic changes in three directions, which has the advantages of high connectivity and easy control of geometric parameters. In recent years, an increasing number of studies have introduced TPMS into stent design [14, 15], among which gyroid (G) and Schwarz (S) are commonly used TPMS stent types. The structure of the G-shaped lattice is closest to that of human bone, and mechanical properties similar to those of cortical bone or cancellous bone can be obtained by adjusting its geometry, so it is one of the most promising bone scaffold structures [16]. S-shaped lattices have greater potential for energy absorption. The normal gyroid lattice porosity increases from 75 to 90%, and when the load angle is adjusted from 90° to 45° relative to the horizontal plane, the stress distribution and plastic strain of the gyroid lattice stent are not greatly affected by the porosity [17]. Some studies have shown that the elastic modulus and yield strength of TPMS lattice structures are better than those of traditional lattice structure scaffolds (e.g., BCC) [15]. To the best of our knowledge, there are few biomechanical studies on porous cervical fusion cages based on different lattice designs, and the biomechanical properties of different types of cages have not yet been completely clarified.

In this study, we propose three new and redesigned porous cervical fusion cages, which may serve as new orthopaedic instruments with lattice structures on the basis of topology optimization, and develop ACDF surgical models, including traditional titanium alloy cervical cages, to comprehensively evaluate the biomechanical properties of different cages.

## Materials and methods

### Establishment of the intact lower cervical spine

The orthopaedic clinic recruited a 27-year-old male volunteer with a height of 173 cm and a weight of 70 kg. The volunteer had no history of medical or surgical diseases and no history of trauma or surgery. Physical examination and X-ray examination excluded acute and chronic cervical spondylosis. Sixty-four rows of CT (GE Healthcare, USA) were performed in the CT room of the Medical Imaging Center of the Fifth People's Hospital of Zhangjiagang (scanning parameters were as follows: layer thickness 0.9 mm, acquisition matrix 512 × 512, pixel size 0.705 mm, field of view 500 mm). The CT scan data were exported and saved in DICOM format, and a total of 276 images were obtained. This research project was approved by the Institutional Review Committee of the Fifth People's Hospital of Zhangjiagang City (L2023001).

**Fig. 1:**
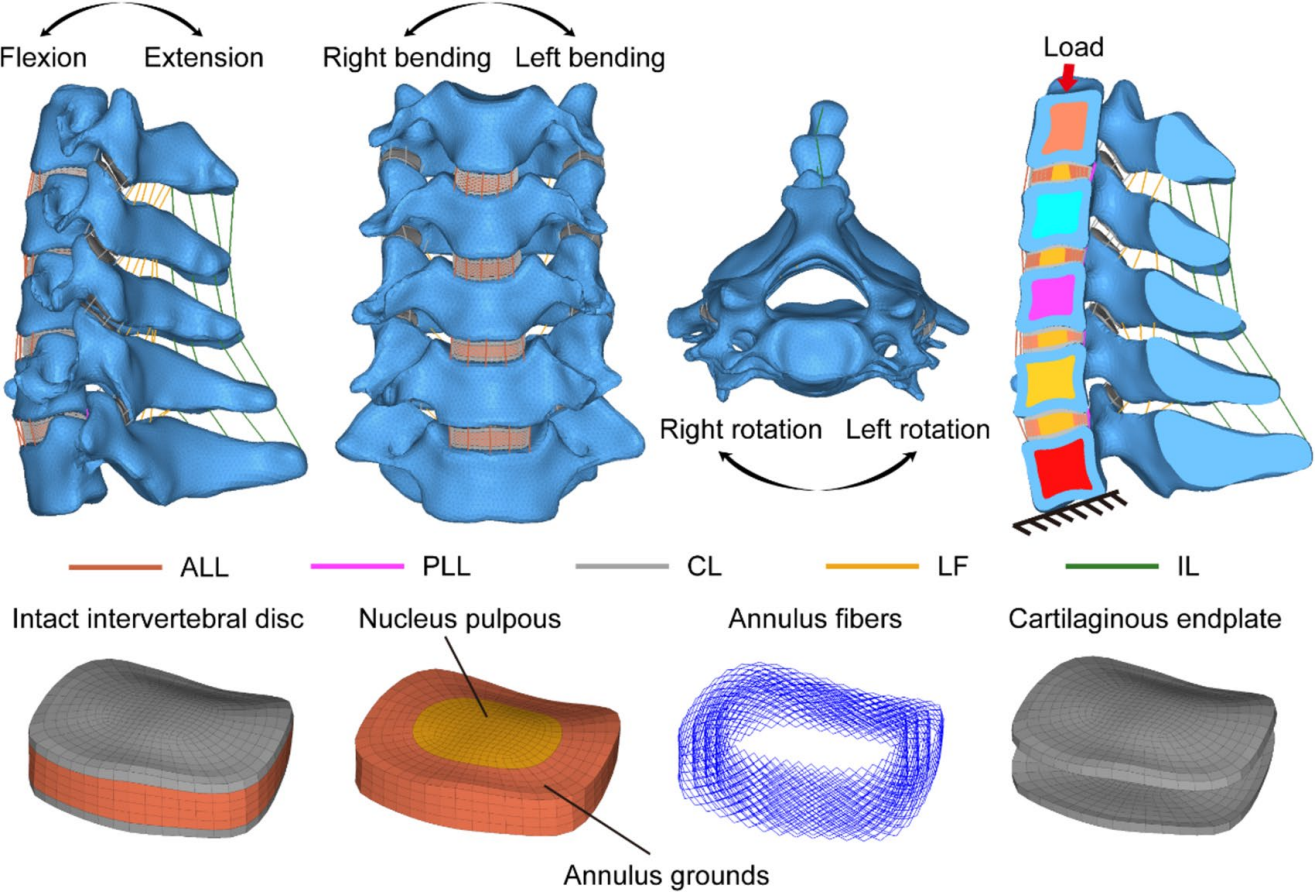
Three basic orthographic views of the complete C3-C7 cervical finite element model and the details of intervertebral disc modeling, showing the schema diagram of bending moments and driven loads in different directions

**Fig. 2:**
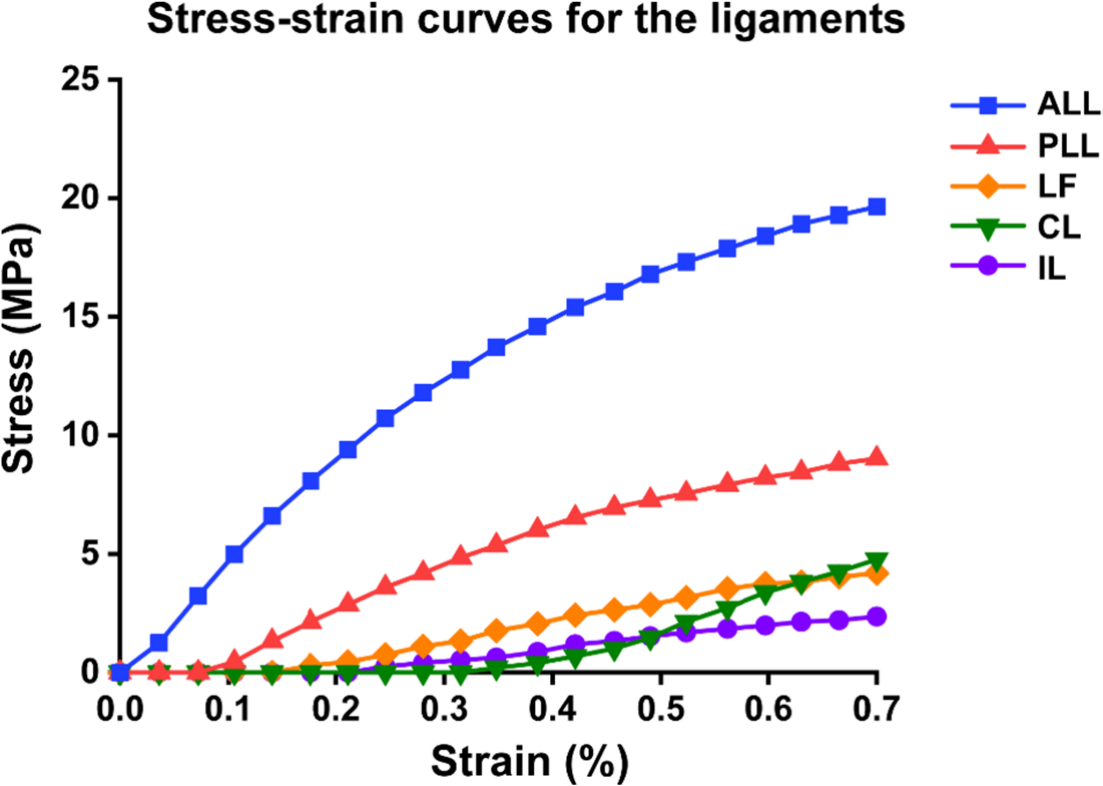
This study investigates the stress–strain relationship properties of ligaments used for finite element modeling. Reprinted with permission from Liu et al. [22, 23]

Based on the functions of threshold segmentation, region growth and manual cutting of Mimics 19.0 (Materialise, Belgium), we built a 3D face mesh model of the C3-C7 segment (STL format). The surface mesh model is imported into the reverse engineering software GeomagicWrap2017 (Geomagic, USA) for modification, such as repair, smoothing, polishing, noise reduction, and constructing surface patches. The IGS format file was imported into the three-dimensional modelling software Pro/E5.0 (PTC, USA), the cortical bone, cancellous bone, intervertebral disc, cartilage endplate and articular process cartilage were reconstructed according to the anatomical structure of cervical vertebrae, and the three-dimensional geometric model of C3-C7 was established. The endplate thickness is set to 0.6 mm. According to literature reports and anatomical data, the annulus fibrosus and nucleus pulposus were segmented according to a 6:4 ratio [18, 19]. The geometric model is then imported into Hypermesh14.0 (Altair, USA) to construct annulus fibrosus and ligaments as well as meshes. Cortical and cancellous bones are meshed with tetrahedral elements, and intervertebral discs are divided into hexahedral grids (as shown in Fig. 1). The divided volume mesh model (INP file) was introduced into Abaqus 6.14 (Dassault, France). Five ligaments were assigned by nonlinear spring materials, namely, ALL (anterior longitudinal ligament), PLL (posterior longitudinal ligament), LF (ligamentum flavum), IL (interspinous ligament) and CL (cystic ligament). The element types and material properties used in the finite element model are shown in Table 1 and Fig. 2, which are based on previous studies [20–24].

**Table 1:** Material properties and mesh type assigned to the FE model

Component	Element type	Material model	Material property	Cross-Sectional Area (mm^2^)
Cortical bone	C3D4	Isoelastic	E = 10000 MPa, v = 0.3	-
Cancellous bone	C3D4	Isoelastic	E = 100 MPa, v = 0.3	-
End plate	C3D8	Isoelastic	E = 5.6 MPa, v = 0.3	-
Facet cartilage	C3D4	Isoelastic	E = 10.4 MPa, v = 0.4	-
Nucleus pulpous	C3D8H	Hyperelastic	C10 = 0.12, C01 = 0.09	-
Annulus fibrosus matrix	C3D8H	Hyperelastic	C10 = 0.133, C01 = 0.0333, D = 0.6	-
Annulus fibers	T3D2	Hypoelastic	E = 350-550 MPa, v = 0.3	-
ALL	T3D2	Nonlinear	Force–deflection curve^a^	6.1
PLL	T3D2	Nonlinear	Force–deflection curve^a^	5.4
IL	T3D2	Nonlinear	Force–deflection curve^a^	13.1
CL	T3D2	Nonlinear	Force–deflection curve^a^	46.6
LF	T3D2	Nonlinear	Force–deflection curve^a^	50.1
Titanium alloy	C3D4	Isoelastic	E = 110000 MPa, v = 0.3	-

*ALL* Anterior longitudinal ligament, *PLL* Posterior longitudinal ligament, *IL* Interspinous ligament, *CL* Capsular ligament, *LF* Ligamentum flavum, *C3D4* Tetrahedron, *C3D8* Hexahedron, *C3D8H* 8-node hybridization unit, *T3D2* Truss, tension only. ^a^Ligament properties are referred to Fig. 2 for details

### Design of cervical porous cages based on topology optimization

**Fig. 3:**
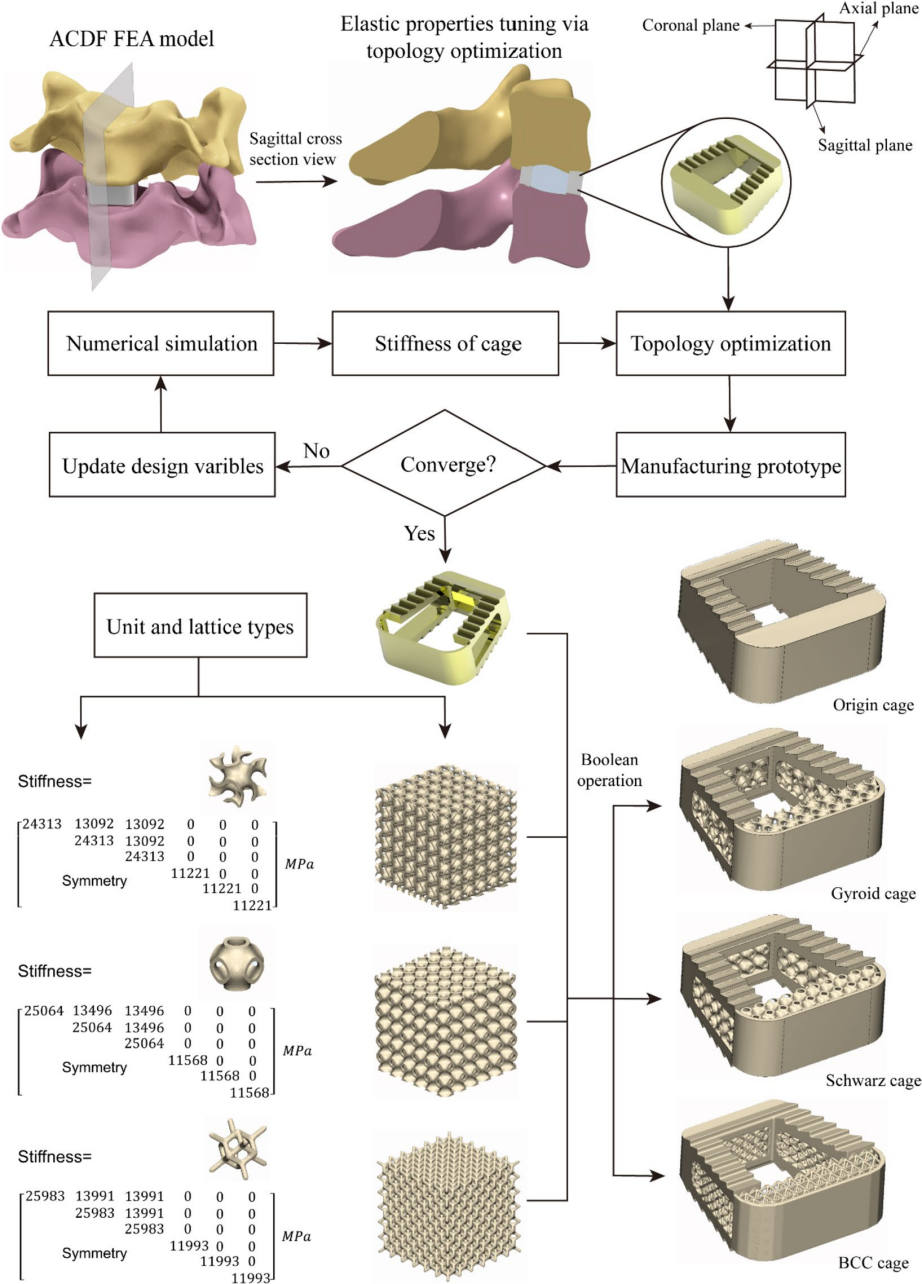
Establish the finite element model of ACDF operation. The flow chart shows the topology optimization of the macro-structure and the parameter design of the cage frame according to the load-bearing results of the high-density fusion cage. Three kinds of porous cervical cages were obtained by filling the frame with different types of lattices

The purpose of the new cage design is to reduce the risk of traditional high-density cage subsidence due to stress concentration and to promote the invasive growth of grafted bone to achieve high-quality fusion between bone and cage. One of the main causes of cage subsidence is known to be a mismatch in the elastic properties between the cage and native bone [25]. Thus, the elastic gradient of the cage is tuned here to achieve a stiffness match to the cortical bone while satisfying the mechanical strength of the device. Therefore, the topology optimization scheme is useful for realizing the lightweight design of the fusion device. Three-dimensional geometric modelling (12 mm × 13 mm × 5 mm, 6° lordotic angle) of the commercially available titanium alloy cage (Cervical Fusion Device 682, Kangli Orthopaedics Instrument, China) was carried out. In contrast to porous structural devices controlled by volume fraction, the original solid titanium alloy cages are known as high-density cages. Figure 3 depicts the workflow for the development of porous cages.

Abaqus6.14 was chosen to optimize the topology of the intervertebral fusion cage. The software employs the SIMP variable density method to establish the relationship between the elastic tensor and the relative density of the unit through the material interpolation model to actualize the structural optimization of the model and find the optimal distribution of structural materials [26]. The goal of global optimization is to minimize the volume of the solid part constrained by stiffness [27]. The mathematical formulation of the macroscopic topology optimization problem is as follows:

$$Objective Function:Minimize ({U}_{c})$$

$$Constraint:0< {\eta }_{i}<1 ( i=\mathrm{1,2},3\dots n)$$

1 $$V\le {V}_{0}-{V}^{*}$$

2 $$V= \sum_{i}{\eta }_{i}{V}^{i}$$

3 $${E}_{i}=E({\eta }_{i})$$

4 $$\{{\sigma }_{i}\}=\left[{E}_{i}\right][\{{\varepsilon }_{i}\}$$

where ${U}_{C}$ is the compliance, ${\eta }_{i}$ represents the internal pseudodensities assigned to each finite element $\left(i\right)$ in the optimization equation, $V$ is the computed volume, ${V}_{0}$ is the original volume, ${V}^{*}$ represents the amount of volume to be removed, ${V}_{i}$ is the volume of element *i*, ${E}_{i}$ is the elasticity tensor for each element, *E* represents the elasticity tensor, ${\sigma }_{i}$ is the stress vector of element *i*, and ${\varepsilon }_{i}$ represents the strain vector of element. *η*, as the density index, ranged from 0 to 1. An *η* value close to 0 indicates that the material is removed, and an *η* value close to 1 indicates that the material is retained. The program was set to reduce the volume by up to 30% and iterate no more than 30 times. The volume fraction of the optimized cage outer frame is 40.3%.

We established a surgical model of ACDF in the C4/5 segment, as shown in Fig. 3. The operation process was as follows: the anterior longitudinal ligament and intervertebral disc of the C4/5 segment were completely resected. After decompression, a cage was implanted in this segment. The Boolean operation removes the excess bone to ensure that the two contact surfaces of the cage are in complete contact with the cortical bone surfaces [28], and the ACDF model was established successfully. Next, five material properties, including cortical bone, cancellous bone and titanium alloy, were assigned according to the information in Table 1. A 1.0 Nm moment was also applied in the FEA models to simulate flexion, extension, lateral bending and axial rotation motion. The follower load of 73.6 N is a physiological compressive load along the physiological curve of the cervical spine to simulate the effect of muscle force and head weight [29]. A reference point was set at the centre of the upper surface of C3, and the reference point was coupled to the upper surface of C3. A pure moment of 1 Nm combined with a vertical load of 73.6 N was applied to the reference point, while the lower surface of C7 was firmly fixed [30]. Set the optimization area, that is, the entire solid cage; set the optimization boundary constraints, that is, C4, C5 and the bone-device contact interface. Under the constraint of a 30% volume fraction, the original cage model was topology optimized to remove redundant units with a relative density of 0, resulting in only the front and rear parts being separated from each other. To maximize the stiffness of the structure, the flexibility of the structure needs to be minimized, so the objective function is set to minimize the structural flexibility. Because the result of topology optimization only outputs the approximate design, the basic trend of optimization is obtained by analysing the results, which guides redesign of the cage. This new cage removes the internal redundant part of the original instrument to obtain the hollowed-out cage frame and obtains the best spatial material distribution through the optimization process. The rigidity of the device is maintained as much as possible under the specified loading conditions, and the reduction in the volume of the solid part increases the space for the bone graft.

To distinguish regular porous structures, common porous structures are divided into two types: one is the strut structure derived from the traditional CAD design idea, which is called the beam structure, and the other is the spatial curved surface structure controlled by mathematical equations, which is called the three-periodic minimal surface (TPMS). Among them, the beam structure commonly used in implants is composed of body-centred cubic (BCC) structures, and the parameter relationship is as follows:

5 $$L= \frac{4\sqrt{3}}{3\mathit{sin}\left(70.5^\circ \right)}\left(\delta +D\right)$$

6 $${V}_{f}=1-\Phi$$

7 $${V}_{f}=K\frac{3\sqrt{3}\pi {D}^{2}}{{L}^{2}}$$

where $L$ is the size of the element, $D$ represents the diameter of the strut, ${V}_{f}$ represents the volume fraction, $\Phi$ is the porosity, and $\delta$ represents the pore value. Because there is solid coincidence at the nodes, the correction coefficient $K$ is introduced, which is generally 0.5–0.6. Porous structures with different performances can be obtained by changing the design parameters of the truss element.

**Fig. 4:**
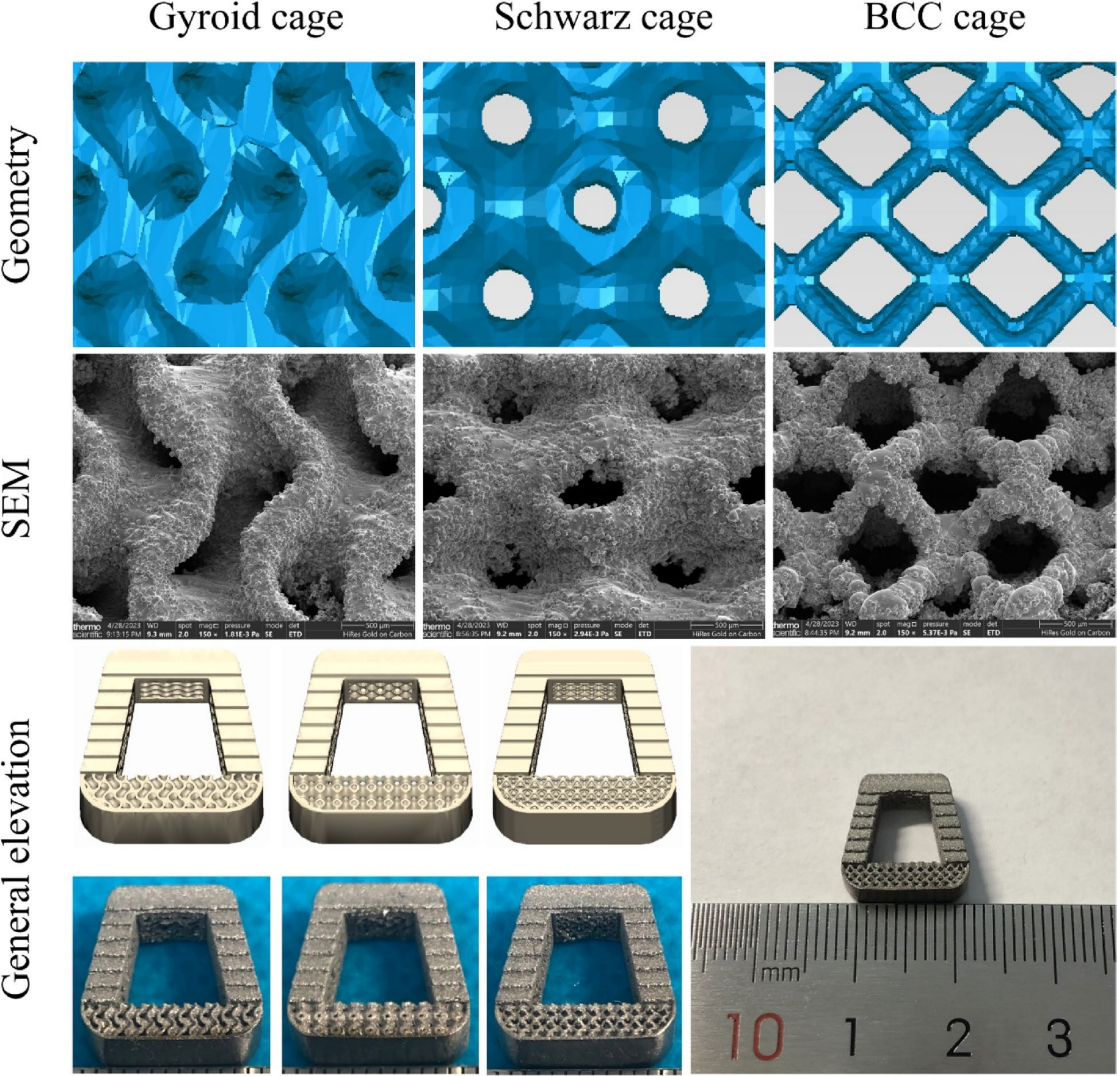
The spatial geometry of the porous cages, 3D printing samples porous structure scanning electron microscopy (SEM) images and macroscopic photographs

TPMS is a general term for curved surfaces with zero mean curvature everywhere on a class of surfaces in three-dimensional space. By adjusting the relevant parameters of the expression of TPMS elements, we can realize the internal and external offset of the element, adjust the structural gap and change the cell period. In this study, we design the structure of the G (gyroid) surface function and S (Schwarz) surface function. The mathematical formulation is as follows:

8 $$G\left(x,y,z\right)=cos\frac{2\pi x}{{L}_{1}}\mathit{sin}\frac{2\pi y}{{L}_{2}}+cos\frac{2\pi y}{{L}_{2}}\mathit{sin}\frac{2\pi z}{{L}_{3}}+cos\frac{2\pi z}{{L}_{3}}\mathit{sin}\frac{2\pi x}{{L}_{1}}+C=0$$

9 $$S\left(x,y,z\right)=\mathit{cos}\frac{2\pi x}{{L}_{1}}+\mathit{cos}\frac{2\pi y}{{L}_{2}}+\mathit{cos}\frac{2\pi z}{{L}_{3}}+C=0$$

where C is a constant that can control parameters such as pore shape, pore size, surface area ratio and porosity, C = 0, and L_1_ = L_2_ = L_3_ = 2π in this study. Previous biocompatibility studies have shown that when the unit size is between 200–400 μm and the porosity is controlled at approximately 60%, bone ingrowth can be promoted [31], while considering that the unit size is too small to cause mesh generation failure and increased computational costs in this study. The target unit cell size is set to 1.5 mm × 1.5 mm × 1.5 mm, and the unit porosity is controlled at 60%. The final porous cages are composed of the optimized design domain reserved part and the nondesign domain predefined frame structure. The Boolean intersection operation between the high-density cage and the porous structure generates the porous structure, and then the Boolean union operation with the framework generates the final porous cages (gyroid cage, Schwarz cage and BCC cage). Figure 4 shows the geometric configurations of the three micropores. Under a scanning electron microscope, it is observed that the micropore structure and geometric structure of the additively manufactured cage samples are highly similar, and a large number of titanium particles are attached to the surface of the device. The macroscopic appearance of the samples and the geometric models have high consistency as well.

### Development of the C4/5 Level ACDF FE model

In the topology optimization section, the single-segment ACDF model of C4/5 was established, and surgical models of different cages were further developed around the research content. According to the type of implanted cages, the models of the high-density cage group (HDCG), gyroid lattice cage group (GLCG), Schwarz lattice cage group (SLCG) and BCC lattice cage group (BLCG) were established. Relatively favourable results have been reported for ACDF surgery using only cages [32]. In this study, CT modelling was performed in a young volunteer. The favourable maintenance of the intervertebral height after the modelling also provides a reason for simplifying the plate fixation. In addition, to reduce uncertainties, the stress distribution after plate implantation interferes with the work of observing the interface stress distribution of different models; therefore, without anterior plate fixation, this FEA project design was used.

### Loading and boundary conditions

Similar to the loading boundary conditions during the topology optimization procedure. For all FE models (including the intact model and four ACDF surgical models), we constrained the C7 lower end plate in all six orientations and set the reference points on the C3 upper endplate. We apply a moment of 1 Nm to the reference point to simulate flexion, extension, lateral bending and axial rotation. Then, we apply a 73.6 N driven load to simulate the weight of the human skull and muscle strength [29]. The contact relation between the facet cartilages is set to face-to-face contact, and the friction coefficient is 0.01 [33]. The bone-cage interfaces were assigned tie constraints to simulate thorough osseointegration [34]. Checking the ROMs of each segment of the intact model in the process compared with previous studies, the validation of the model can be verified.

### Convergence test

**Fig. 5:**
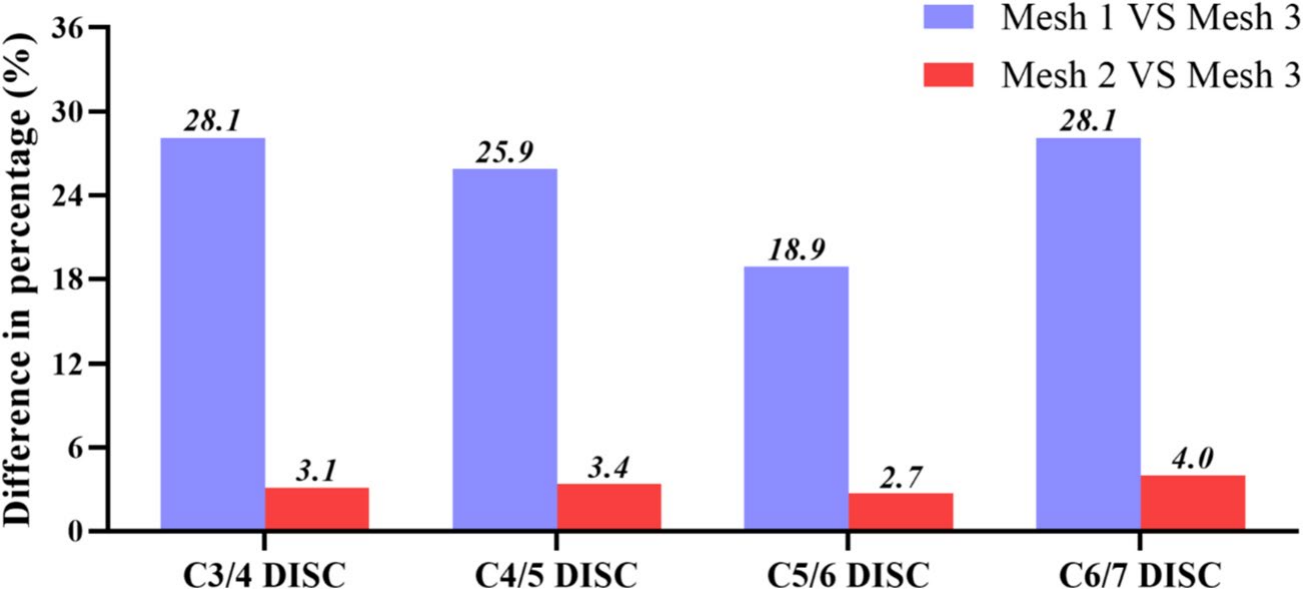
Percentage differences of von Mises stress for each segment of the intervertebral disc of FEM model at three grid sizes

We used the intact model for convergence testing and generated three mesh resolutions based on different grid sizes. Information on the elements and nodes for each mesh resolution is shown in Table 2. The maximum stress of each segment of the intervertebral disc was measured when a preload force of 73.6 N was applied at the C3 reference point. The stress differences among the three types of mesh intervertebral discs were compared. Taking mesh3 as a reference, the maximum stress difference of each segment of the intervertebral disc in mesh2 is less than 5% (as shown in Fig. 5), which has favourable convergence and can be used for further research [35]. The unit size range is 0.8–1.5 mm.

**Table 2:** Element and node numbers for three different mesh resolutions

	Element number	Node number
Mesh 1	64,477	16,345
Mesh 2	288,045	76,080
Mesh 3	413,250	96,419

## Results

### FE model validation

**Fig. 6:**
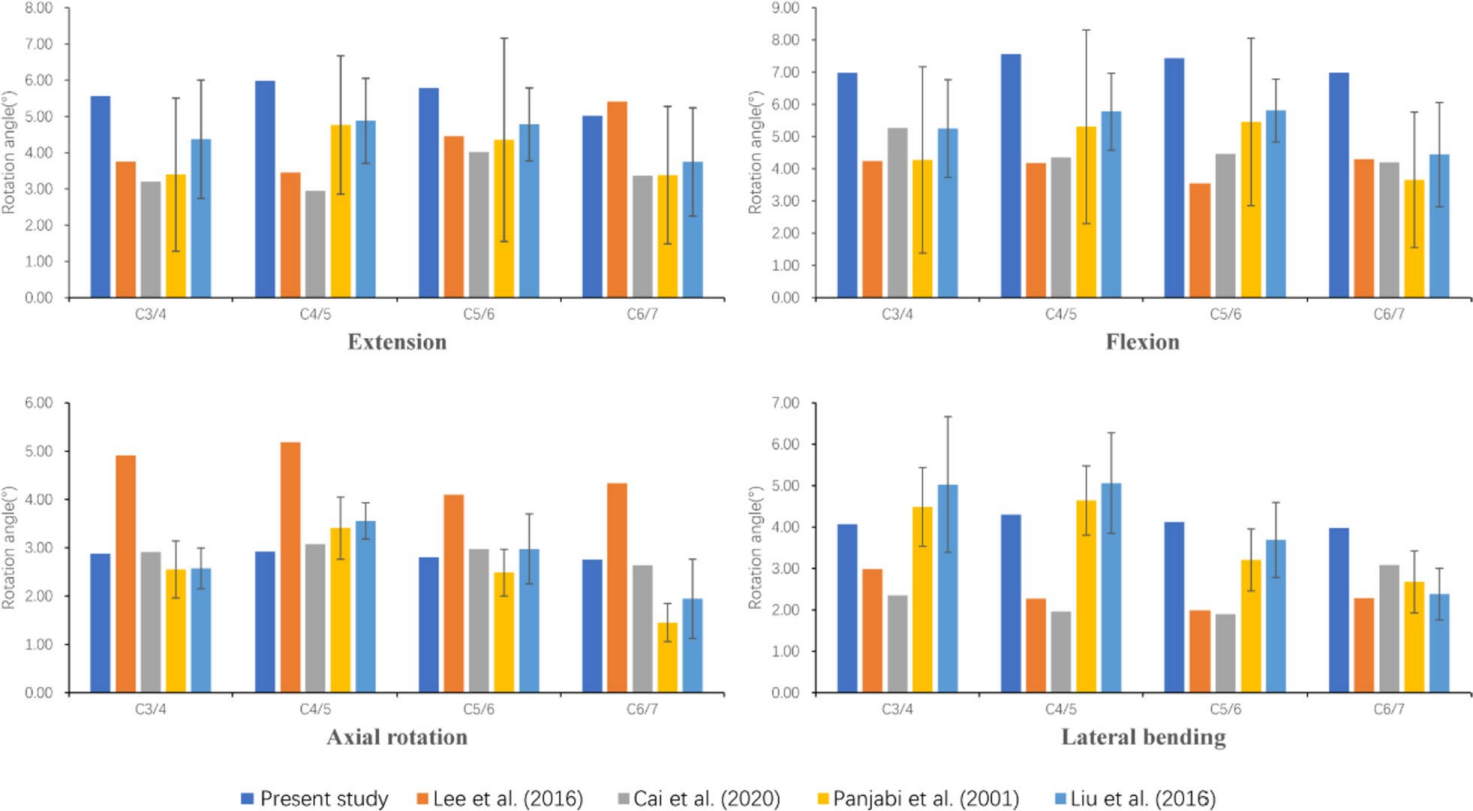
Comparison of the intersegmental ROMs (°) with those in previous studies, and validity has been proven

During finite element analysis of the intact model (IM), we obtained the range of motion (ROM) for multiple cervical segments, and the ROMs for extension of C3-4, C4-5, C5-6, and C6-7 were 5.56°, 5.98°, 5.79°, and 5.02°, respectively; for flexion, these values were 6.98°, 7.56°, 7.43°, and 6.99°, respectively; for axial rotation, they were 2.88°, 2.92°, 2.80°, and 2.76°, respectively; and for lateral bending, they were 4.07°, 4.30°, 4.12°, and 3.97°, respectively. As shown in Fig. 6, the ROMs of each direction of motion demonstrated favourable consistency compared to previous studies, proving the effectiveness and applicability of finite element modelling in this application [36–39].

### Facet contact force

**Fig. 7:**
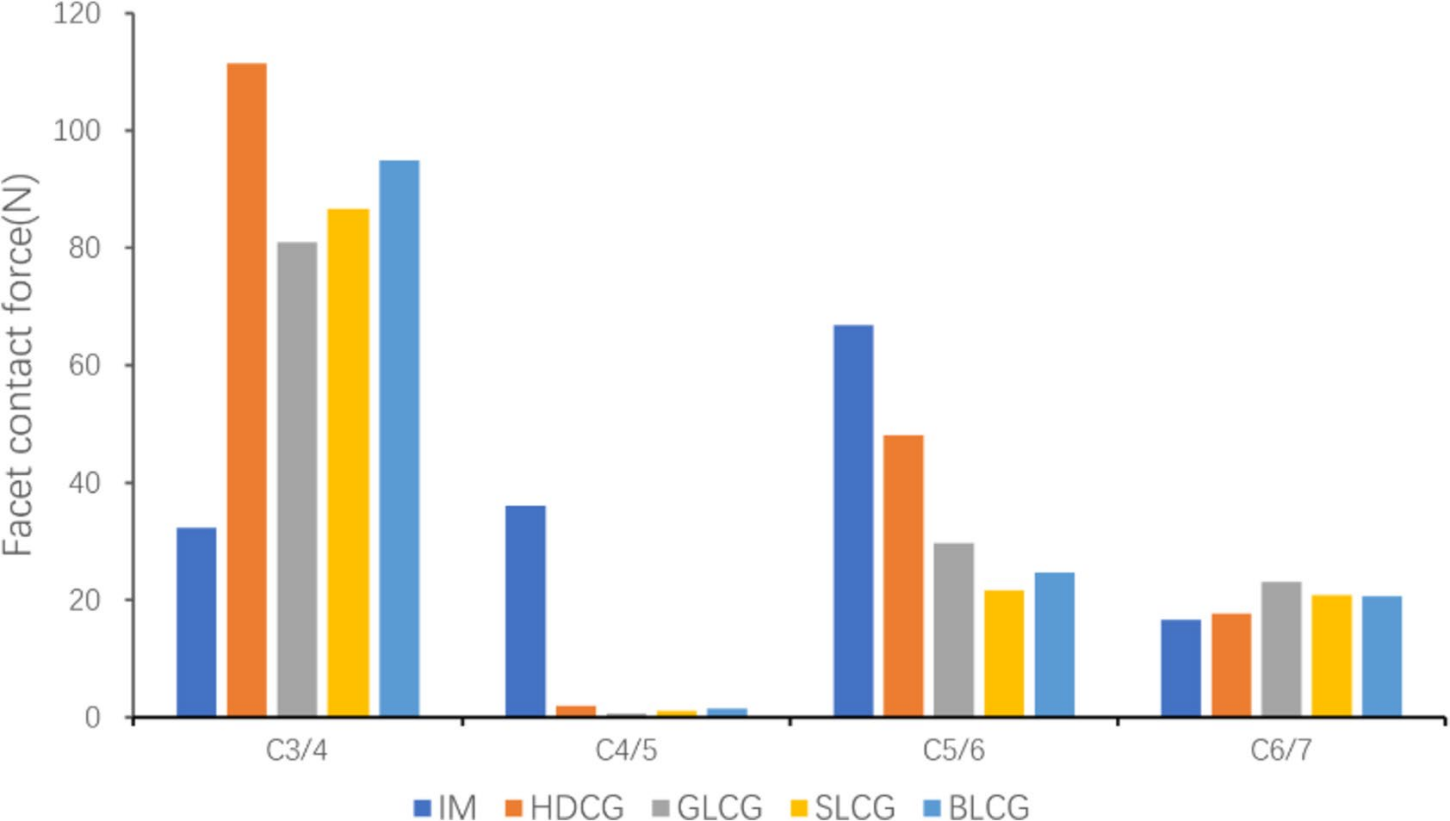
Facet contact force in extension

As shown in Fig. 7, comparing the facet contact force (FCF) of the intact model and the surgical models shows that the FCF of the intact model reaches the maximum value at the C5/6 segment and has little change in the remaining segments, thus showing a trend of increasing and then decreasing in value. All surgical models were fused with the C4/5 segment, so the FCF was almost 0 at the segment, and the C3/4 segment performed functional compensation against the action of the extension moment. The FCF values of HDCG, GLCG, SLCG and BLCG increased by 244.8%, 150.4%, 168.1% and 193.8%, respectively, compared with that of the intact model. In addition, the FCF value of the HDCG model was higher than that of the GLCG, SLCG and BLCG at the C3/4 and C5/6 segments, indicating that the porous cages played a positive role in improving the stress conduction of the facet joints.

### Intradiscal pressure

**Fig. 8:**
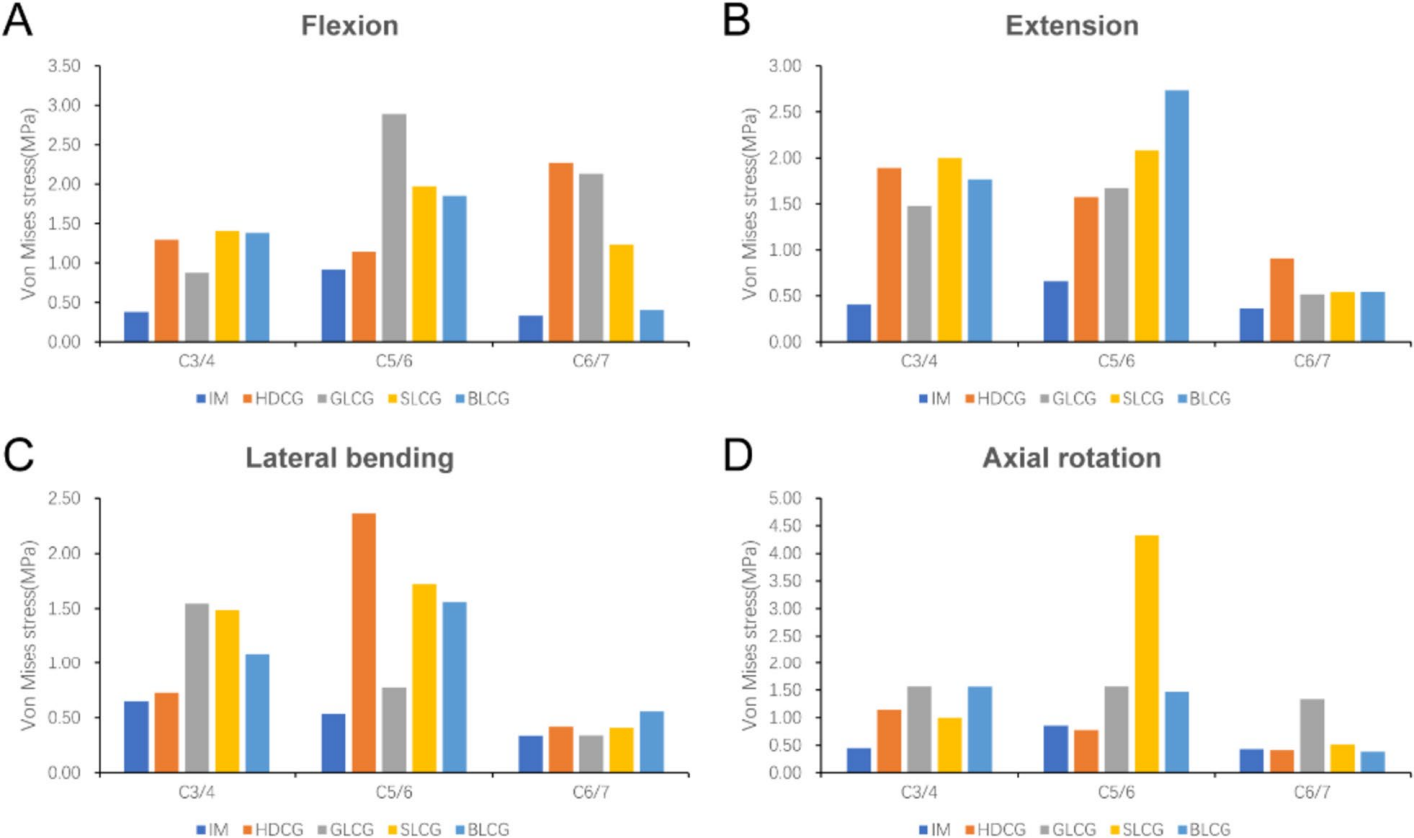
Maximal Von Mises stress at non-surgical segments during different conditions in (**A**) flexion, (**B**) extension, (**C**) lateral bending, and (**D**) axial rotation

The results of intradiscal pressure (IDP) measurements at the nonsurgical segments are shown in Fig. 8. The IDP values ​​of the four types of cervical cages were higher than those of the intact model in flexion, extension, lateral bending and axial rotation, and ACDF surgery increased the IDP values ​​of the adjacent segments. The maximum value of IDP in the 4 surgical models all appeared in the C5/6 segment. Among these values, GLCG reached the maximum value of 2.89 MPa in flexion, BLCG reached the maximum value of 2.73 MPa in extension, HDCG reached the maximum value of 2.36 MPa in lateral bending and SLCG reached the maximum value of 4.33 MPa in axial rotation, reflecting that different types of cages show no clear advantage in conducting load through adjacent segments.

### Stress at the cortical bone interfaces

**Fig. 9:**
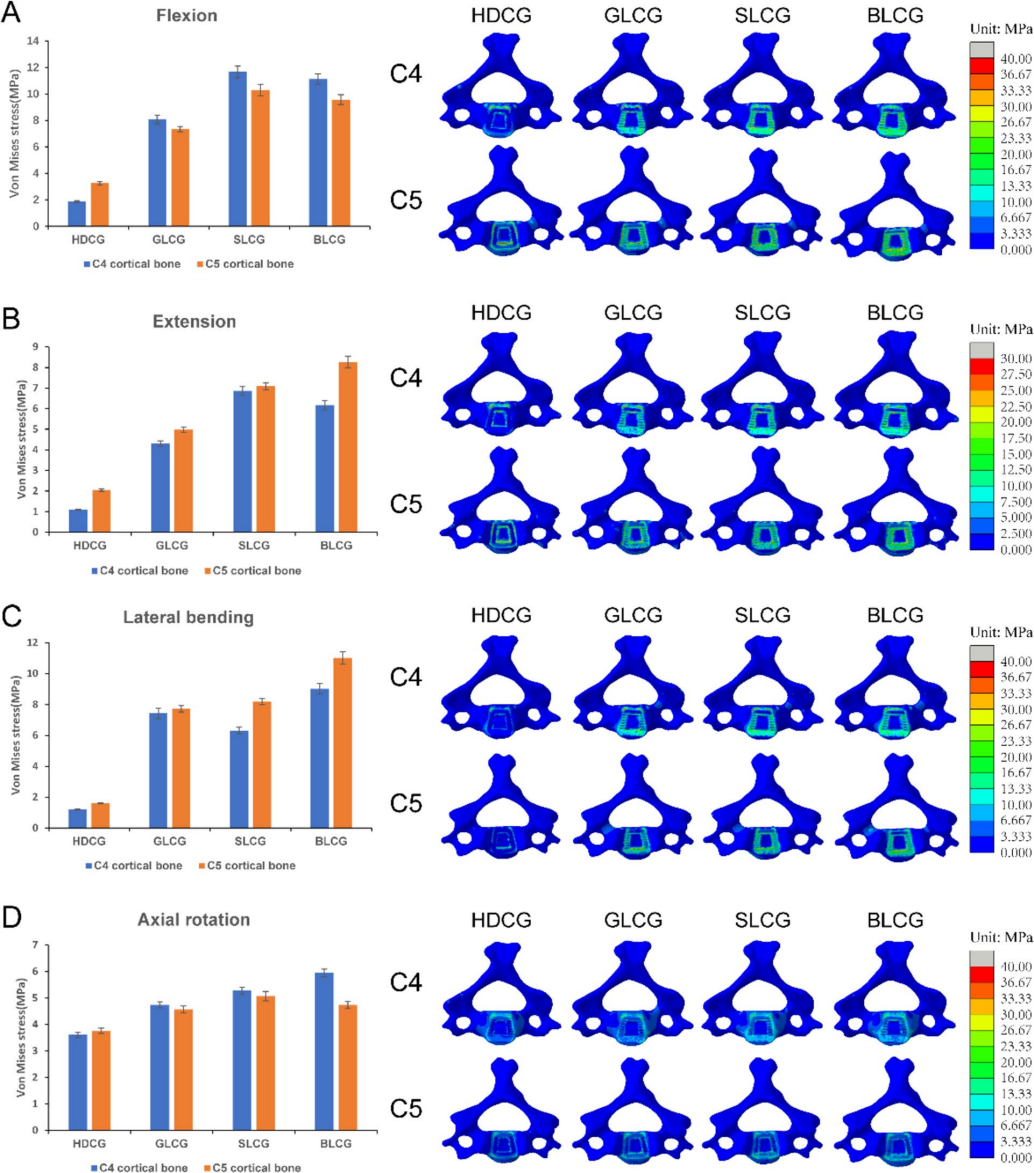
Comparison of the average von Mises stress at the contact interface of C4 and C5 cortical bone in HDCG, GLCG, SLCG and BLCG models, and the corresponding C4 and C5 stress cloud maps during different conditions in (**A**) flexion, (**B**) extension, (**C**) lateral bending, and (**D**) axial rotation

The average von Mises and contour maps of C4 and C5 during flexion, extension, lateral bending, and rotation for the four surgical models are shown in Fig. 9. The numerical data show that the cortical bone surface average stress of the three groups of porous fusion models is higher than that of the high-density fusion group surgical model. Under flexion conditions, the cortical bone average stress of C5 for the GLCG, SLCG, and BLCG models increased by 125.8%, 216.3%, and 194.4%, respectively, compared to that of the HDCG model. Similar phenomena were observed in the other three working conditions. The stress contour maps show that the stress of the HDCG model is concentrated at the inner and outer edges of the bone-cage contact interface under different loading environments, while the stress distribution of the GLCG, SLCG, and BLCG models is relatively uniform. The equivalent stress contour map of the GLCG group cortical bone presents a light green distribution under different conditions, with a smaller coverage area of contour lines than that of the SLCG and BLCG models, and the cortical bone average stress of the GLCG group is the smallest among the three surgical models, reflecting the favourable potential of the gyroid cage in improving stress concentration and preventing fatigue fractures.

### Stress at the cage interfaces

**Fig. 10:**
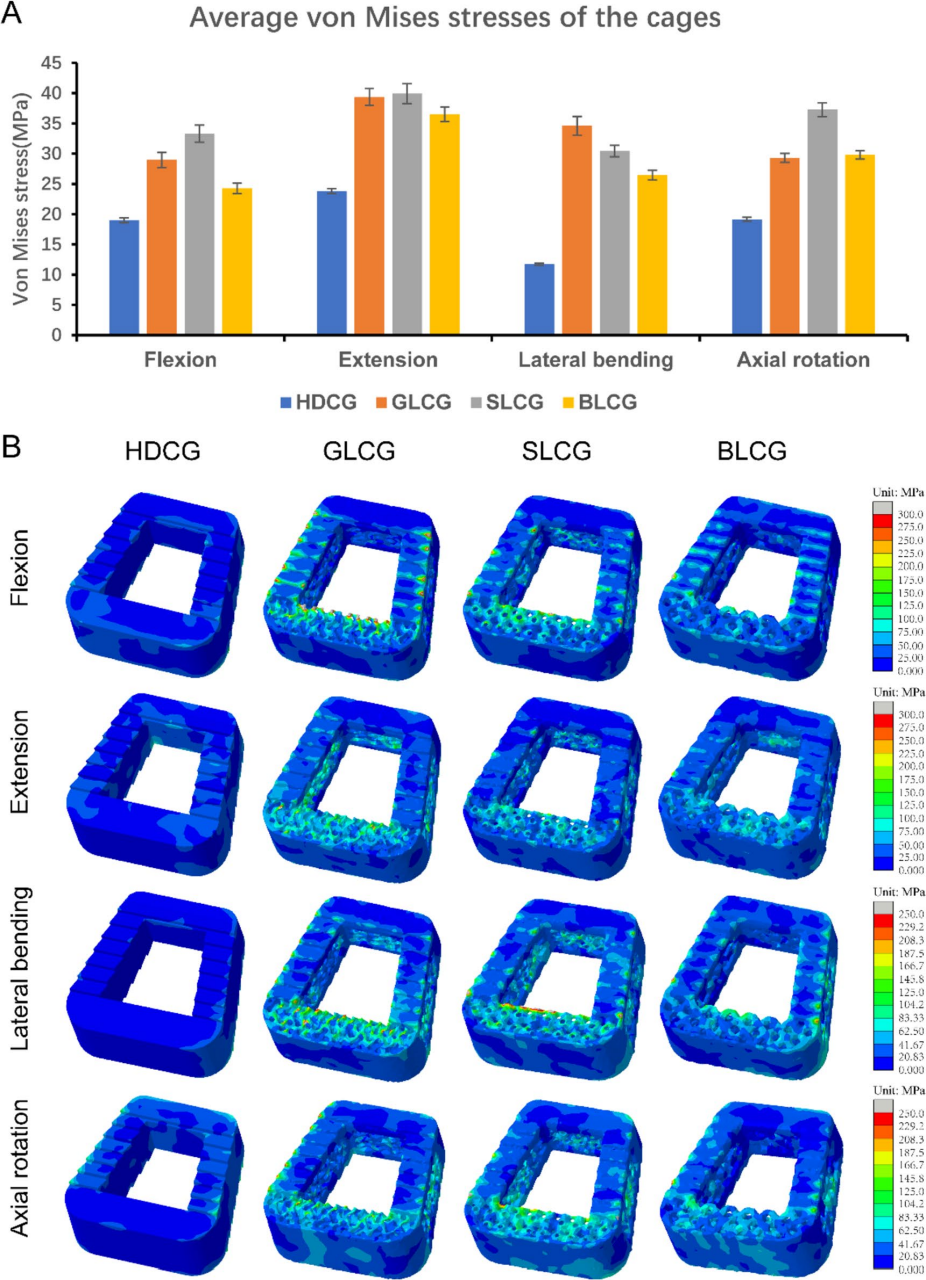
Comparison of the mechanical properties of four kinds of fusion cages under different conditions (**A**) the average stress produced; (**B**) the stress distribution on the surface of different types of cages

Figure 10 shows the average stress and stress contour maps of the four fusion devices under different loading conditions. Similar to the results presented in Fig. 9, the cortical bone stress of the porous fusion device group was generally higher than that of the HDCG group in most environments. Based on the mechanical performance of the three porous fusion devices under all conditions, the Schwarz cage has the highest average stress, followed by the gyroid cage, and the BCC cage has the lowest stress. Under lateral bending conditions, the stress differences among the three porous cages and solid cage are maximized. The average stress of the gyroid cage, Schwarz cage and BCC cage exceeds that of the solid cage by 194.8%, 159.2% and 125.1%, respectively. The stress contour maps show that the high gradient stress contour lines on the surfaces of the gyroid cage and Schwarz cage cover more areas than those of the high-density fusion device and the BCC cage, and the high-stress area is concentrated in the lattice-filled area. The stress distribution on the surface of the high-density fusion device is similar to that of the cortical bone stress distribution, which is concentrated at the inner and outer edges. The lower stress level exhibited by the BCC cage is beneficial for obtaining more durability under complex working conditions than the other two porous fusion devices, indicating that the BCC cage based on beam structure design has more advantages in stability.

## Discussion

The main outcome of this study is that three novel porous neck cages are proposed by combining macroscopic topology optimization and micropore design. Compared with traditional high-density cages, these porous cages have better microstructure and appropriate stiffness, which facilitates bone ingrowth and reduces the risk of stress shielding and cage subsidence. ACDF is shown to be an effective surgical method for the treatment of cervical spondylosis. After resection of the cervical intervertebral disc, support material needs to be placed in the intervertebral space. The implant used in the early stage is an autologous three-sided cortical bone iliac bone block, which has the advantages of no rejection and favourable fusion. However, autologous ilium bone extraction not only prolongs the operation time but also causes certain injuries and complications, and the incidence of complications can reach 20% to 25% [40]. To avoid cutting the ilium, anterior cervical reconstruction materials such as intervertebral fusion cages and hydroxyapatite artificial bone blocks have appeared to replace iliac bone blocks, among which PEEK and titanium alloy intervertebral fusion cages are the most widely used. Due to the mismatch of material stiffness and the inert isolation of high-density materials, the contact surface between the fusion cage and the vertebral endplate cannot achieve the effect of close contact and match, which may lead to complications such as micromovement and subsidence of the implant [41, 42]. A novel design concept is thus adopted, including redesigning the outer frame of the fuser according to the effect of topology optimization at the macro level and filling the optimized gaps with TPMS lattices or BCC lattice at the micro level, and the three designed porous cages demonstrate the mechanical strength, corrosion resistance and biocompatibility of titanium alloy (Ti-6Al-4 V), as well as a lower elastic modulus than titanium alloy, which is beneficial to improve the stress shielding of the contact surface [12]. Its porous structure, similar to bone, provides favourable conditions for bone ingrowth.

We measured the facet contact force (FCF) of multiple models under extension movement. The results showed that the facet joint force of the C3/4 segment was significantly higher than that of the normal model in both the high-density fusion cage group and the porous fusion cage group, while the FCF value of the operation group tended to 0 in the C4/5 operation segment, indicating that the segment was rigidly fused without relative motion. This phenomenon has been confirmed by several finite element studies [37, 43]. The joint force of C4/5 is compensated by the upper adjacent segment. Interestingly, the joint load at the C5/6 segment decreased in the operation group and returned to a normal value at the C6/7 segment. The FCF of the upper adjacent segment was more affected by fusion than the lower adjacent joint. Comparing the FCF of different types of fusion cages, it can be found that in the C3/4 and C5/6 segments affected by fusion, the FCF of the porous cage groups is smaller than that of the high-density cage group, indicating that the former has the ability to potentially protect the facet joints of adjacent segments. Measurement of IDP is crucial to assess changes in adjacent segmental stress. Increased intradiscal pressure in adjacent segments of the operated segment after ACDF may be related to the development of adjacent segment degeneration, and increased IDP in adjacent segments after surgery may be associated with discogenic lesions and subsequent pain [44]. Surprisingly, the low-stiffness porous cages did not appear to improve stress overload in the adjacent segmental disc in the current study.

Hamai et al. [45] reported that a radiolucent area can be observed under the prosthesis after TKA, and we propose that this area is caused by stress shielding to cause bone resorption, which eventually leads to local bone mass decline. To comprehensively evaluate the performance difference between the porous cage and the high-density cage, various stress test environments were established in our finite element analysis. This indicates that the load can be transferred to the bone more uniformly to reduce stress shielding, and the low stiffness of the porous structure helps to improve the interfacial contact state. Mechanical stress is an important factor in osseointegration, but excessive loading is one of the main causes of fatigue damage [46]. In this study, we noticed that in the porous cage groups, the cortical bone stress values ​​of GLCG were the lowest in flexion, extension, and rotation conditions, which achieved a uniform stress distribution at the expense of lower stress feedback. The potential of porous cages to improve the behaviour of the bone-device interface does not stop there. Eventually, bone grows into the pores on the upper and lower surfaces of the cage, and the stress on the endplates may be further reduced with the increased contact area [47]. Therefore, from the perspectives of mechanics and osseointegration, porous cages reduce the risk of subsidence and increase the fusion rate.

Lattice materials are ideal for biomedical applications because the porous structure close to bone can be used to fabricate tough metal instruments by additive 3D printing [48]. Traditional lattices are lattice beam systems composed of intersecting nodes and struts or shells consisting of repeating cells at the base. High strength and low weight characteristics are important advantages of this type of structure, and the lattice structure reduces the stress mismatch with cortical bone, thereby minimizing stress shielding effects and supporting bone growth. Simultaneously, these structures provide nutrient channels and assist oxygen diffusion between the bone tissue and the implant to promote bone development [49]. Combined with the cervical porous cages developed here, we expounds the potential value of this research for promoting bone growth: (i) the porous structure can simulate the pore structure of natural bone tissue and contribute to the attachment and growth of bone cells and (ii) increase the contact area between the implant and the bone tissue in the implant area. The fusion cage can better combine with the surrounding bone tissue and promote the occurrence of bone fusion. (iii) The porous structure can provide channels to promote the growth and diffusion of new blood vessels. Favourable blood supply and adequate nutrients meet the growth and metabolic needs of osteocytes. (iv) A porous structure can reduce the stress mismatch and stress shielding effect between the implant and the surrounding bone tissue. This can reduce the risk of nonfusion and improve the success rate of bone fusion.

Recently, with the deepening of the concept of lattice design, people have gradually paid attention to TPMS lattices. The specific surface area of ​​TPMS scaffolds is high, which is more suitable for cell adhesion and growth [50]. The growth of bone cells in porous scaffolds requires mechanical load stimulation, and a certain range of compressive loads and fluid shear stresses can stimulate bone cell growth [51]. The perfusion system can provide compression and fluid load simultaneously. Some studies have achieved favourable results in vitro cell culture through this device [52], showing favourable biocompatibility of the TPMS lattice. Studies have shown that the elastic modulus and yield strength of TPMS scaffolds are better than those of traditional lattice structure scaffolds [15]. Compared with TPMS lattices with smooth surfaces, cubic lattices with sharp corners show higher stress concentrations [53]. However, in this study, after observing the Mises cloud images, we found that the area of ​​the high-stress area on the surface of gyroid-cage and Schwarz-cage was larger than that of BCC-cage, and the results of comparative analysis of the average stress also found the same phenomenon. Several important variables affect the design of lattice structures, including cell structure, cell size, relative density, and bulk porosity. These variables greatly affect the mechanical properties and density of the created structures [54]. In addition, reviewing the modelling process of these porous cages, to improve the calculation efficiency, a coarser mesh is used, resulting in irregular thin walls that can cause local stress concentration, which weakens the advantage of dispersing stress on the regular surface of the TPMS lattice. Overall, whether porous cages made of TPMS lattices or BCC lattices have the ability to improve the stress mismatch of the contact interface, the prospects of TPMS cages with higher requirements for design and manufacturing processes in the field of biomedical applications are better; nevertheless, they are also confronted with the challenges of structural strength and cost, and the overall optimal design of porous cages still needs to be systematically tested.

Finite element analysis is shown to be an effective method to predict the changing trend of different implants and provide certain guidance for device development. However, this study has certain limitations. First, idealized experimental conditions were used, and the study focused on the stress performance of the C4/5 segmental fusion, which may not cover all cases. Second, to enable the fusion cage to fully bear the stress load, and thus to better observe the distribution of stress on the surface of the fusion cage, we simplified the steps of plate fixation and set the fusion cage and the cortical bone surface as tie constraints to simulate complete fusion. Any possible micromotion is thus neglected. Third, while various porous cages with bone-like trabecular structures were designed, the optimized model lacks experimental verification and provides trends instead of actual data. The next step of the research plan is to arrange compression, shear, and fatigue tests to compare and verify the numerical simulation results. There may be differences between the theoretical model of the porous structure of bone trabecula and the real test piece after 3D printing in terms of structural size, including pore diameter and wall thickness. The current modelling data should therefore be interpreted with caution.

## Conclusion

In this study, topology optimization techniques and porous materials were integrated to redesign titanium alloy cervical fusion cages to improve their biomechanical properties. The optimized porous cage shows a satisfactory and low elastic modulus, which reduces the risk of cage subsidence and stress shielding. The porous structure not only increases the space for bone grafting but also provides interconnecting channels for bone ingrowth, which is more conducive to osseointegration. The optimization proposed here provides reference value for future research and development of new orthopaedic devices.

### Supplementary Information

**Additional file 1. **

## Data Availability

The datasets used and/or analyzed during the current study are available from the corresponding author upon reasonable request.

## Abbreviations

ACDF: Anterior cervical discectomy and fixation operation
TO: Topology optimization
TPMS: Three-periodic minimal surface
BCC: Body-centered-cubic
G: Gyroid
S: Schwarz
ALL: Anterior longitudinal ligament
PLL: Posterior longitudinal ligament
LF: Ligamentum flavum
IL: Intervertebral ligament
CL: Cystic ligament
CAD: Computer Aided Design
SIMP: Solid Isotropic Material with Penalization
IM: Intact model
HDCG: High density cage group
GLCG: Gyroid lattice cage group
SLCG: Schwarz lattice cage group
BLCG: BCC lattice cage group
FCF: Facet contact force
IDP: Intradiscal pressure
